# Structure-based virtual screening towards the discovery of novel thrombin inhibitors with Anti-HCC activities

**DOI:** 10.3389/fchem.2024.1451574

**Published:** 2024-09-20

**Authors:** Xiaoxi Zhang, Xumei Zheng, Chun Han, Lintao Wu

**Affiliations:** ^1^ Department of Mental Health, Changzhi Medical College, Changzhi, Shanxi, China; ^2^ Department of Chemistry, Changzhi University, Changzhi, Shanxi, China

**Keywords:** thrombin, hepatic carcinoma, computer-assisted drug design, molecular docking, *in vitro* experiment

## Abstract

**Introduction:**

Hepatic carcinoma (HCC) is one of the most lethal malignant tumors in the world, and new treatment regimens for this disease are urgently needed. Studies have shown that thrombin stimulates tumor progression by forming fibrin and activating platelets. Dabigatran etexilate, a thrombin inhibitor, can inhibit the activity of thrombin and prevent the proliferation and metastasis of HCC in cells and nude mice.

**Methods:**

The present study was designed to find thrombin inhibitors with novel skeletons, and further confirm the correlation between thrombin inhibition and HCC prevention to identify potential anti-HCC drug leads.

**Results and Discussion:**

The potential thrombin inhibitors were firstly screened in the Topscience Database, and 20 potential active molecules were found by molecular docking. The effect of these molecules on thrombin inhibition, coagulation and tumor proliferation were evaluated, and the definite activity of ZXX-4 was identified. Further *in vivo* assays in nude mice showed that ZXX-4 inhibited tumor proliferation in nude mice, reduced tumor metastasis, and enhanced the clinical efficacy of first-line drug sorafenib for the treatment of HCC. ZXX-4 can be further explored as an anti-tumor lead compound with a novel skeleton, and inhibition of thrombin can serve as a potential treatment strategy for HCC.

## 1 Introduction

Coagulation factor IIa, also known as α-thrombin or thrombin for short, is a serine protein hydrolase synthesized in the liver through the activation of plasminogen. Its structural and catalytic characteristics are similar to those of pancreatic trypsin, a proto-typical digestive serine protease (Troisi et al., 2021). In 1992, Bode et al. elucidated the crystal structure of thrombin, revealing it as a protein comprising two chains linked by a disulfide bond with a molecular weight of 37 kDa: a light chain A composed of 39 amino acid residues and a heavy chain B composed of 259 amino acid residues (Bode et al., 2008). Thrombin serves as the main effector protease in primary hemostasis by facilitating platelet activation (Posma et al., 2016). Thrombin is produced at the site of vascular injury following activation of plasminogen, subsequent thrombin triggers activation of other clotting factors (V, VIII, XII) and platelets, leading to platelet aggregation at the site of vascular injury (Versteeg et al., 2013). Finally, thrombin converts nearby plasma fibrinogen to fibrin, interwoven fibrin causes platelets and blood cells to coagulate to form a thrombus, ultimately stopping bleeding (Mackman et al., 2007). Recent studies have demonstrated thrombin directly modulates tumor behavior by activating platelets, promoting tumor aggregation, and facilitating tumor adhesion to the subendothelial stroma (Hu et al., 2004; Nierodzik and Karpatkin, 2006). In addition, serine protease-activated receptors (PARs) can act as sensors to indirectly trigger various responses in the tumor and stroma. Among them, PAR-1, PAR-3, and PAR-4 can be activated by thrombin, and PAR-2 can be the target of a variety of serine proteases (e.g., trypsin, trypsin-like enzymes, granzyme A, and coagulation factors). However, PAR-1 is usually predominant in tumors and is thought to contribute to cancer progression and metastasis (Elzer et al., 2008). Numerous studies have shown that venous thromboembolism (VTE) is an important cause of morbidity and mortality in cancer patients (Key, 2019). As early as 1865, scientist Armand Trousseau observed a relationship be-tween idiopathic venous thromboembolism and underlying cancer driven by thrombin (Alexander and Gilmour, 2022). A comprehensive Danish study conducted in the general population in recent years has revealed a steady increase in the risk of venous thromboembolism among cancer patients. The cumulative incidence of venous thromboembolism at 12 months in cancer patients was nearly ninefold higher than in noncancer subjects (2.3% vs 0.3%) (Mulder et al., 2021). The etiology of cancer-associated thrombosis is multifactorial and results from the interaction of cancer-related factors (e.g., hypercoagulability due to cancer procoagulants, release of thrombogenic factors, systemic inflammation), patient-related factors (age, gender, individual immune response to cancer), and hospitalization- or treatment-related factors (chemotherapy and immunotherapy, central venous catheterization, surgery, braking) (Patnaik and Moll, 2008).

Antithrombin (AT) is a potent inactivator of thrombin and a major inhibitor of blood coagulation, AT physiologically inactivates thrombin (factor IIa) and factor Xa (FXa). The main anticoagulant drugs currently used in clinical practice include indirect thrombin inhibitors such as polymeric heparin, vitamin K antagonists, and coumarin, as well as direct thrombin inhibitors such as argatroban, ximelagatran, melagatran, efegatran, and dabigatran etexilate (Dab). This family of inhibitors inhibits not only thrombin but also thrombin-mediated factors V, VIII, and XII activity (Dong et al., 2016). Dab (Pradaxa^®^), developed by Boehringer-Ingelheim in Germany, is a well-studied and promising direct oral thrombin inhibitor. Dab, as a prodrug of dabigatran, was among the first direct oral anticoagulants, approved for use in the European Union for patients under 18 years old and in the United States for patients aged 3 months to less than 18 years old (Paik, 2022). Overall, Dab has demonstrated effectiveness and a favorable safety and tolerability profile (Paik, 2022; Ferro et al., 2019).

**FIGURE 1:**
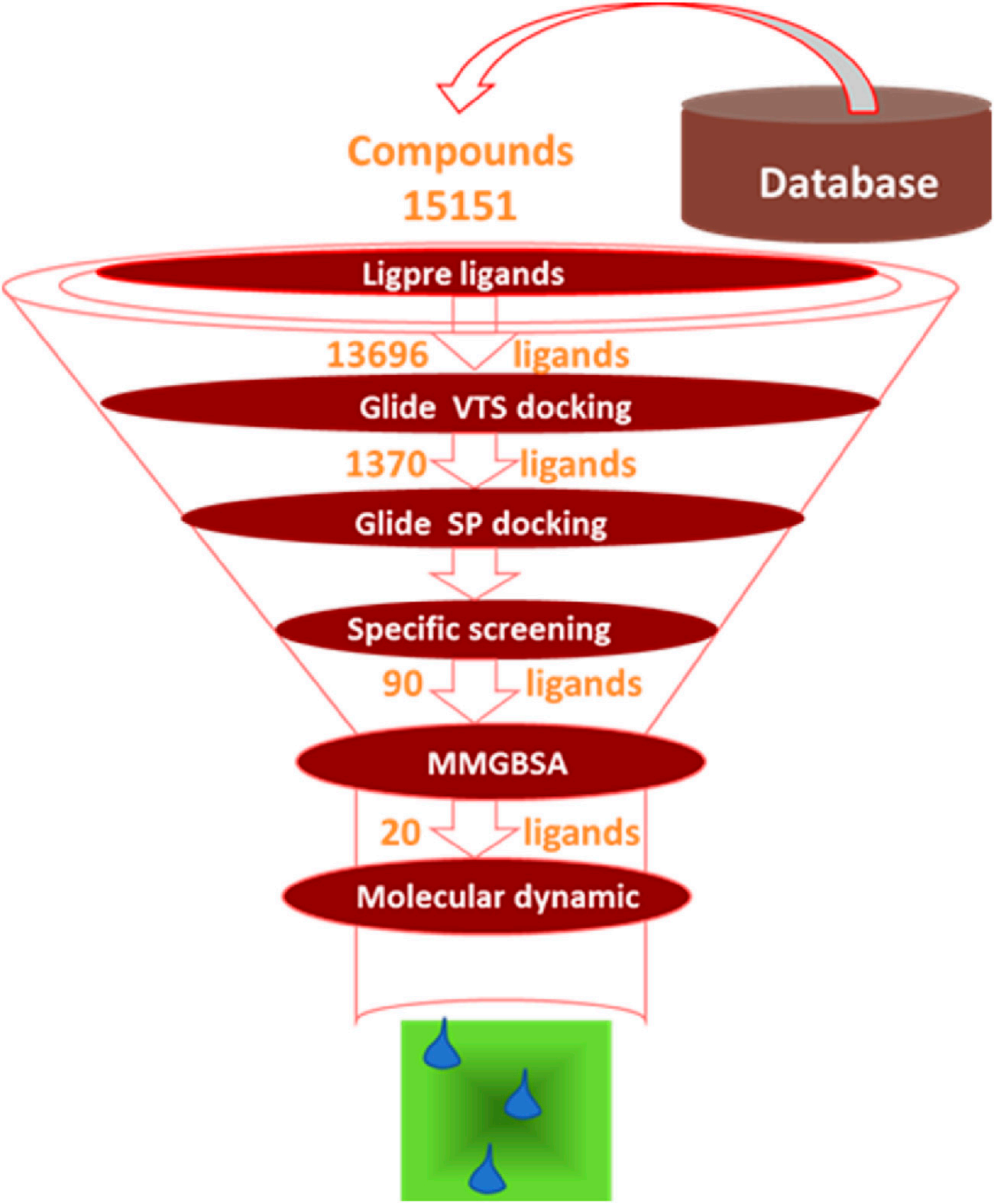
Small molecules screening process.

Hepatocellular carcinoma (HCC) is the most common primary liver cancer and ranks as the sixth most common malignant tumor worldwide. It poses challenges in prevention, diagnosis, and treatment and has a high recurrence rate after surgery, posing a significant threat to human life and health (Sung et al., 2021). Currently, there are three main types of immuno-therapy for liver cancer: immune checkpoint inhibitors, liver cancer vaccines, and cellular therapies. Among these, immune checkpoint inhibitors show promising results in clinical trials. HCC evades antitumor immune responses by expressing ligands in tumor and mesenchymal cells, making immunotherapy a hopeful treatment option (Chen and Flies, 2013). Studies have shown that thrombin is significantly overexpressed in HCC tissues and positively correlates with cancer cell metastasis (Xue et al., 2010). Xie et al. designed and synthesized a series of dabigatran derivatives based on the direct thrombin inhibitor dabigatran. Through docking studies, they identified a precursor drug, BX-2, which exhibited potent inhibitory effects on thrombin-induced platelet aggregation and proliferation of HCC cells (Figure 1). In addition, the drug BX-2 reduced tumor volume, weight, lung metastasis, and secondary tumorigenesis in a nude mouse model. Combination therapy with sorafenib further enhanced the efficacy of BX-2. This study lays the foundation for elucidating the mechanism of action of liver cancer drug targets and contributes to the advancement of liver cancer drug research (Xie et al., 2024).

In this study, we aim to discover novel thrombin inhibitors and validate the therapeutic potential of thrombin inhibition for HCC by combining computer-assisted drug de-sign screening, thrombin inhibition screening assays, and anti-HCC activity evaluation.

## 2 Materials and methods

### 2.1 Preparation of the protein molecule

The structure of thrombin was selected from the PDB database (PDB ID: 1KTS), with a resolution of 2.40 Å. The crystal structure represented the X-ray crystal structure of the thrombin complex with the positive compound. Schrodinger’s protein preparation wizard (2021) was used to add hydrogen, and assign charge and protonation state at pH 7.0, and OPLS-2005 force field was used to minimize the structure (Davie and Kulman, 2006; Chobanian et al., 2015). In the study by Schrodinger, the prepared protein was used to prepare the protein structure, which mainly included removal of water molecules, loop remodeling, and side chain and hydrogen atom optimization, as shown in the diagram. Next, Glide 6.6 module was used to generate the grid, with the positive compound structure selected as the center, and the grid frame defined as 20 × 20 × 20 Å. 15,151 molecules in the database were used as screening sources. In Schrodinger’s study, all compounds were imported into the Ligprep 3.3 module to prepare ligands, as described below: Epik 3.1 was used to generate the ionized state of the molecules at pH 7.0 ± 2.0, and then stereoisomers were generated. The generated grid and prepared ligands were used for docking-based virtual screening using Glide 6.6.

### 2.2 *In vitro* thrombin inhibition assay

Compounds ZXX-1 to ZXX-20 were dissolved in DMSO and diluted to different concentrations. Lyophilized thrombin, which was purchased from China National Institute for the Control of Pharmaceutical and Biological Products, was reconstituted (5.4 μg/mL), added to the diluted solution, and pre-incubated at 37°C for 10 min. Next, specific fluorogenic thrombin substrate III Ac-FVR-AMC (5 μM) was added in each group, and fluorescence was measured 20 times every 15 s for about 10 min using a microplate reader with an excitation wavelength of 355 nm and an emission wavelength of 460 nm. The dynamic changes of fluorescence intensity were detected by using dabigatran as a reference drug. Three experiments were conducted for each assay, and the inhibition rate and 50% inhibitory concentration (IC_50_) of thrombin were calculated using the following formula:

$$\text{Inhibition rate }\left(\%\right)=\left[\text{VDMSO }‐\text{ Vsample}\right]/\text{VDMSO}\times 100\;\%V$$

sample: initial velocity of compound group; VDMSO: initial velocity of blank group, treated with DMSO alone; Inhibition rate (%) = 100/[1 + 10 (LogIC_50_−X)h], h represents Hill coefficient.

### 2.3 Inhibitory effect on thrombin-induced platelet aggregation *in vitro*

Blood was collected from rats and centrifuged for 10mins to obtain platelet-rich plasma, which was centrifuged again to collected platelets. The platelets were resus-pended and diluted to a concentration of 5 × 10^5^/mL. PRP (250 mL), and thrombin (2.5 mL) were added to the platelet suspension in each group and incubated at 37°C. After incubation for 120 s, the reaction solution was well-mixed, and the same amount of thrombin (2.5 mL) was added to a final concentration of 0.2 U/mL. The platelet aggregation rate and inhibition rate were calculated as follows:

$$\text{Aggregation rate sample }\left(\%\right)=\text{the moving of recorder since the addition of thrombin}/60\times 100\%$$

$$\text{Inhibition rate of aggregation sample }\left(\%\right)=\left[\text{aggregation rate }\left(\text{CMC}‐\text{Na}\right)‐\text{aggregation rate }\left(\text{Sample}\right)\right]/\text{ aggregation rate }\left(\text{CMC}‐\text{Na}\right)\times 100\%$$

### 2.4 Tables cell culture and proliferation assay

Cell lines Hep3B, HepG2, HCCLM3 and HUh7 were purchased from Shanghai Biolaf Biotechnology Co., Ltd., and certified by short tandem repeat profiling and *mycoplasma* and cell vitality testing. The proliferation rate was determined using a cell proliferation assay kit (TOPSCIENCE, C0005). Briefly, cells were seeded in 96-well plates at a concentration of 10,000 cells/well. MTS was added and incubated at 37°C for 2 h. The absorbance was measured at a wavelength of 490 nm. Three independent experiments were per-formed for each assay.

### 2.5 BALB/c nude mice

Animal experiments were approved by the Animal Ethics Committee of Chinese Academy of Medical Science (Permit No. IMB-20190423D702), and all procedures were carried out in accordance with the guidelines of the Animal Care and Use Committee of Chinese Academy of Medical Science. 20,000 HepG2 cells were injected into the fat pad of each female Balb/c mouse. The long and short diameters of the tumor were measured using a caliper. Medication was started when a rumor size of 100 mm^3^ was reached. Sorafenib, ZXX-4, and sorafenib combined with ZXX-4 were administered for 28 days. The body weight and tumor size of the mice were measured twice a week. The mice were sacrificed after 28 days of treatment, the tumor weight was measured, and the number of metastases was counted by Indian ink staining of the lung.

## 3 Results and discussion

### 3.1 Molecular docking screening of small molecules and thrombin protein

**FIGURE 2:**
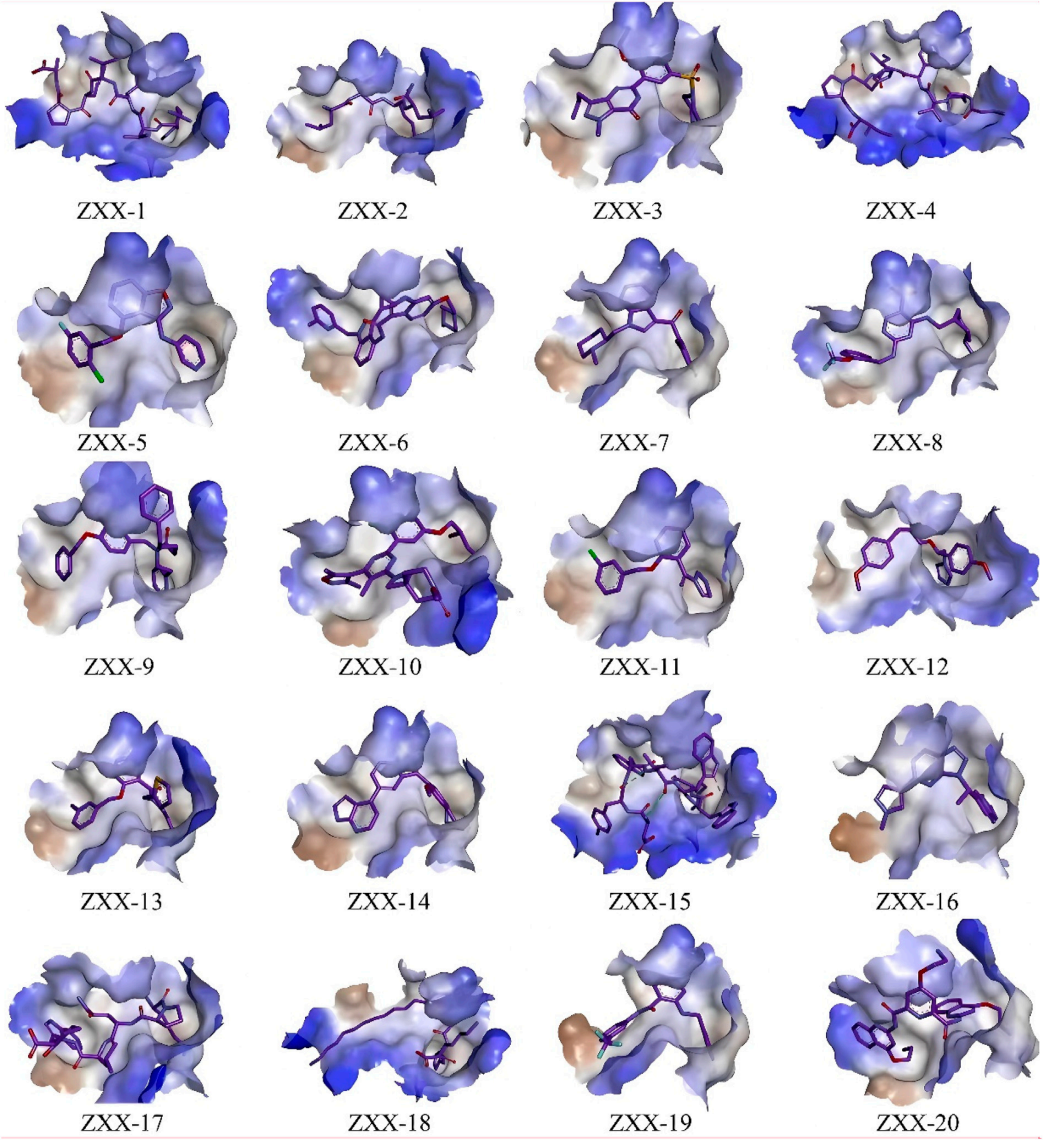
Binding pattern of selected compounds to thrombin protein.

**FIGURE 3:**
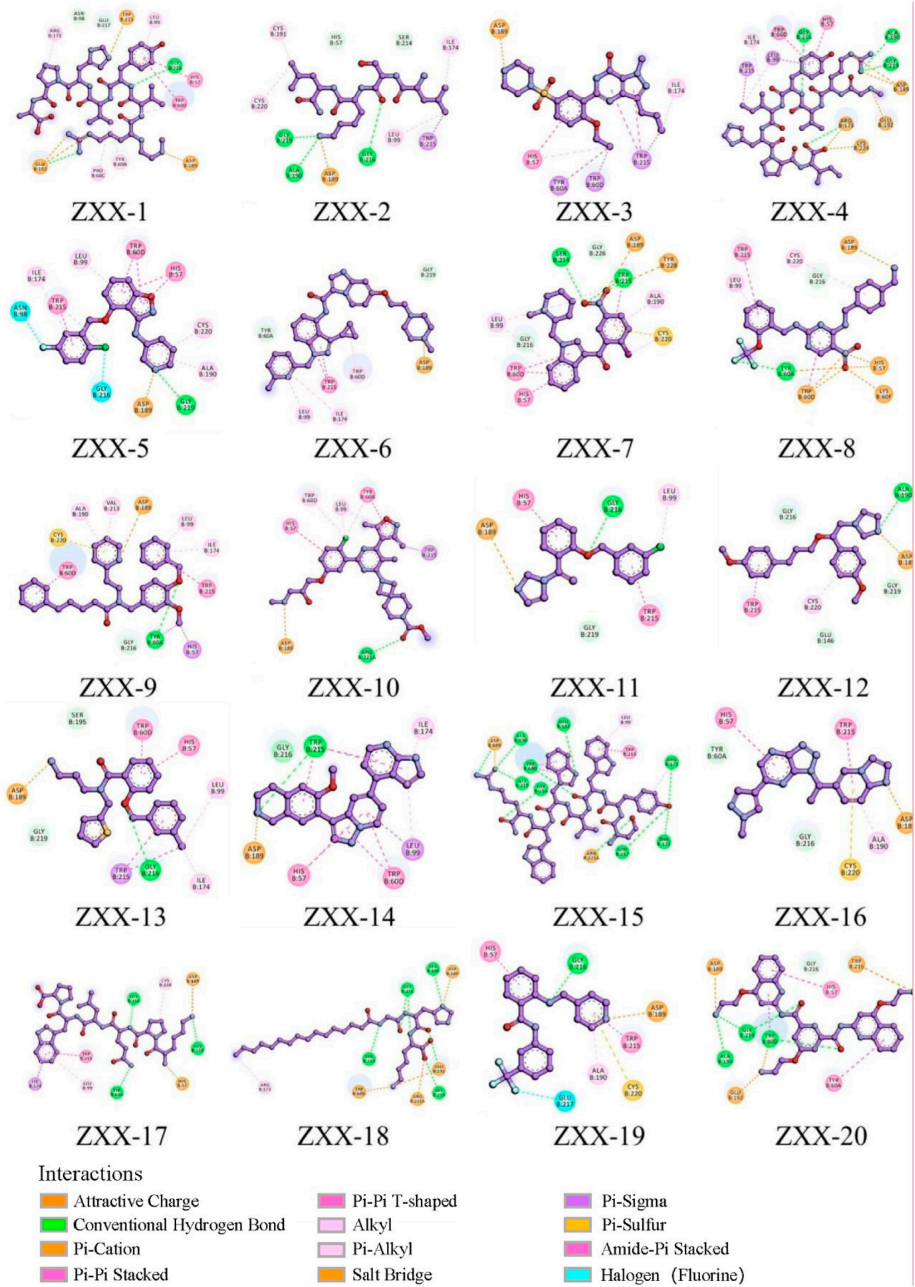
The interaction of the compound with the key amino acids of thrombin.

The crystal structure with an interaction between thrombin and dabigatran (PDB ID: 1KTS) was selected, with a resolution of 2.40 Å. The protein was firstly optimized using the Schrödinger platform (2021). The receptor grid was then generated using the Glide 6.6 module, and the center of the ligand was defined based on the study of Xie et al. A library of 15,151 compounds was used as the screening source and imported into the Ligprep 3.3 module to prepare ligands. Virtual screening based on molecular docking was conducted using Glide 6.6, and the process was shown in Figure 1. Firstly, Glide/HTVS was used for rapid high-throughput screening, and the top 10% molecules were selected for subsequent screening. Next, Glide SP molecular docking was then performed, and amino acids, in-cluding ASP189, TRP215, and GLY216, were focused according to the reported interaction patterns. Finally, the binding energy between ligands and thrombin was screened using MM-PBSA, and 20 molecules with the highest affinities were selected for further investigation. The structure and binding energy of selected compounds ZXX-1 to ZXX-20 were presented in Table 1, the binding mechanism between small molecules and thrombin was presented in Figure 2, and the key amino acids involved in the binding between small molecules and thrombin were presented in Figure 3.

**TABLE 1:** Scoring of selected compounds.

NO	Name	CAS	MM-GBSA	Charge	Favorable	Favorable	Glide
ZXX-1	Saralasin acetate	39698-78-7	−76.3755	1	3	2	−9.03022
ZXX-2	LSKL	N/A	−69.6818	1	2	1	−9.29927
ZXX-3	N-Desmethyl Sildenafil	139755-82-1	−69.3465	2	5	1	−7.7587
ZXX-4	[Sarl, Ile8]-Angiotensin II acetate	N/A	−68.0807	1	2	3	−10.7707
ZXX-5	SMS2-IN-2	2241838-28-6	−67.8163	1	3	3	−8.69688
ZXX-6	ARRY-382	1313407-95-2	−67.5977	1	4	1	−7.57856
ZXX-7	AM1241	444912-48-5	−67.4525	1	4	5	−8.22648
ZXX-8	PKC-theta inhibitor	736048-65-0	−66.6726	2	2	4	−10.1253
ZXX-9	MCP110	521310-51-0	−66.3957	1	2	2	−8.29124
ZXX-10	EZM 2302	1628830-21-6	−66.3589	1	3	1	−8.01711
ZXX-11	CROCONAZOLE	77175-51-0	−65.7003	1	3	2	−7.42095
ZXX-12	SKF-96365 hydrochloride	130495-35-1	−65.0600	1	4	2	−7.46927
ZXX-13	AMTB hydrochloride	926023-82-7	−63.9699	1	4	3	−8.93964
ZXX-14	PKCiota-IN-2	2230055-52-2	−63.9156	1	6	1	−8.32117
ZXX-15	MEN 10207 acetate	N/A	−63.5550	1	3	2	−12.3219
ZXX-16	Savolitinib	1313725-88-0	−63.3812	1	2	2	−9.00551
ZXX-17	C-Reactive Protein (CRP) 201–206 acetate	N/A	−62.0821	1	2	2	−8.68713
ZXX-18	PalMitoyl Tripeptide-1 Acetate	1628252-62-9	−62.0351	2	1	3	−8.15129
ZXX-19	AAL-993	269390-77-4	−60.7397	2	1	1	−6.75672
ZXX-20	Pyridostatin Trihydrochloride	N/A	−60.2356	1	2	2	−8.72258

### 3.2 Thrombin and blood coagulation inhibitory activity *in vitro*

Inhibitory activities of these 20 selected compounds on thrombin were evaluated *in vitro* using dabigatran as a control. As shown in Table 2 compounds had an IC_50_ of less than 200 nM for thrombin, and ZXX-4 was identified as the most active compound among others with an IC50 of 0.023 μM, which was still weaker than dabigatran (with an IC_50_ of 0.007 μM).

**TABLE 2:** Thrombin inhibitory activity of **ZXX-1** to **ZXX-20**.

NO.	IC_50_ (μM)	NO.	IC_50_ (μM)
**ZXX-1**	0.175 ± 0.018	**ZXX-11**	0.072 ± 0.020
**ZXX-2**	0.068 ± 0.012	**ZXX-12**	0.083 ± 0.016
**ZXX-3**	N/A	**ZXX-13**	0.123 ± 0.018
**ZXX-4**	0.023 ± 0.005	**ZXX-14**	N/A
**ZXX-5**	0.045 ± 0.010	**ZXX-15**	N/A
**ZXX-6**	N/A	**ZXX-16**	N/A
**ZXX-7**	N/A	**ZXX-17**	0.052 ± 0.009
**ZXX-8**	N/A	**ZXX-18**	N/A
**ZXX-9**	N/A	**ZXX-19**	N/A
**ZXX-10**	N/A	**ZXX-20**	0.098 ± 0.015
		**Dabigatran**	0.007 ± 0.002

**FIGURE 4:**
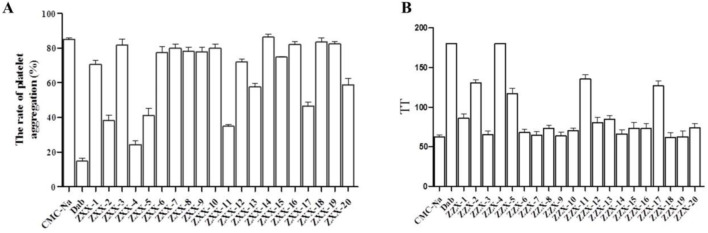
Anticoagulant activity of ZZX-1 to ZXX-20. **(A)** Inhibitory effect on thrombin-induced platelet aggregation; **(B)** Thrombin time. Concentration of drugs: 3 mg/mL; Con-centration of thrombin: 0.2 U/mL.

**FIGURE 5:**
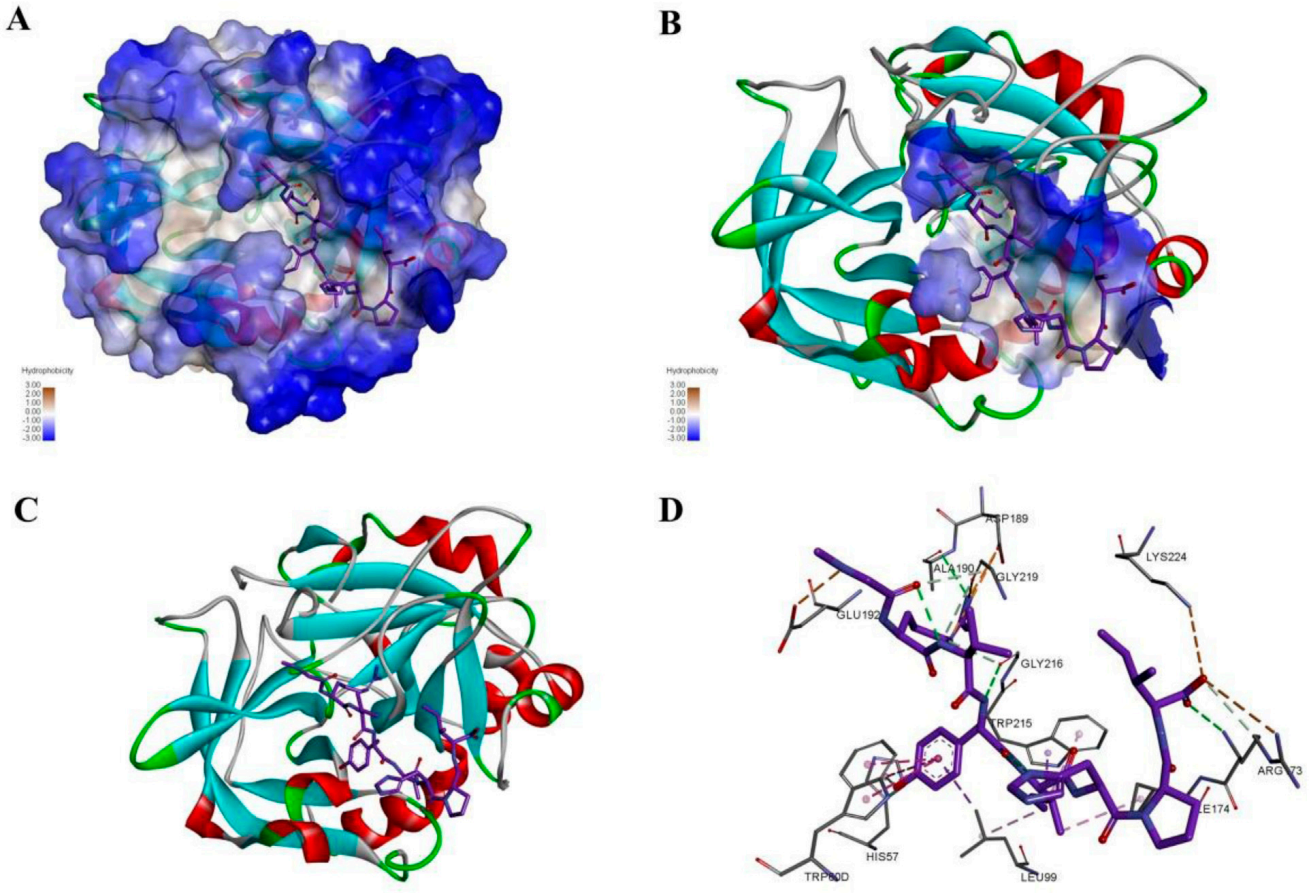
The interaction mode and binding position of compound ZXX-4 with thrombin. **(A)** Hydrophobic surface demonstrating the structure of F2 and the binding of compounds, where blue color represents hydrophilic structural domains and white color represents hydrophobic structural domains; **(B)** demonstrates the location of chemotactic binding to protein structural domains, where the structural domains are shown with hydrophobic surfaces; **(C)** with cartoon representation of F2 protein binding to compounds, where red represents alpha helices, blue represents β folding, and white represents irregular curling; **(D)** diagram indicates the specific three interacting amino acids, green indicates hydrogen bonds, orange indicates salt bridges, and pink indicates PI-Alkyl conjugate interactions.

The effects of these compounds on thrombus-induced platelet aggregation and thrombin time were further evaluated, and the results were presented in Figures 4A, B. Compared with the blank control group, platelet aggregation rate was less than 50% in the positive control dabigatran etexilate group and the ZXX-2, ZXX-4, ZXX-5, ZXX-11, and ZXX-17 groups, with significant inhibitory effect on coagulation. However, the platelet aggregation rate of all compounds tests was higher than that of the positive control dabigatran etexilate, and the difference between ZXX-4 and dabigatran etexilate was not significant. The activities of these compounds to prevent blood clotting were evaluated by measuring thrombin time, and it was found that dabigatran etexilate and 5 compounds, including ZXX-2, ZXX-4, ZXX-5, ZXX-11, and ZXX-17, significantly prolonged the throm-bin time. In fact, both ZXX-4 and dabigatran etexilate prolonged the thrombin time to 180 s (the upper limit of detection). The position of the ligand in the active pocket is shown in Figures 5A–D. Therefore, these 5 compounds were selected to evaluate their inhibitory effect on thrombin-induced accelerated tumor proliferation *in vitro*.

### 3.3 Inhibitory effect of compounds on thrombin-induced accelerated proliferation of HCC

**FIGURE 6:**
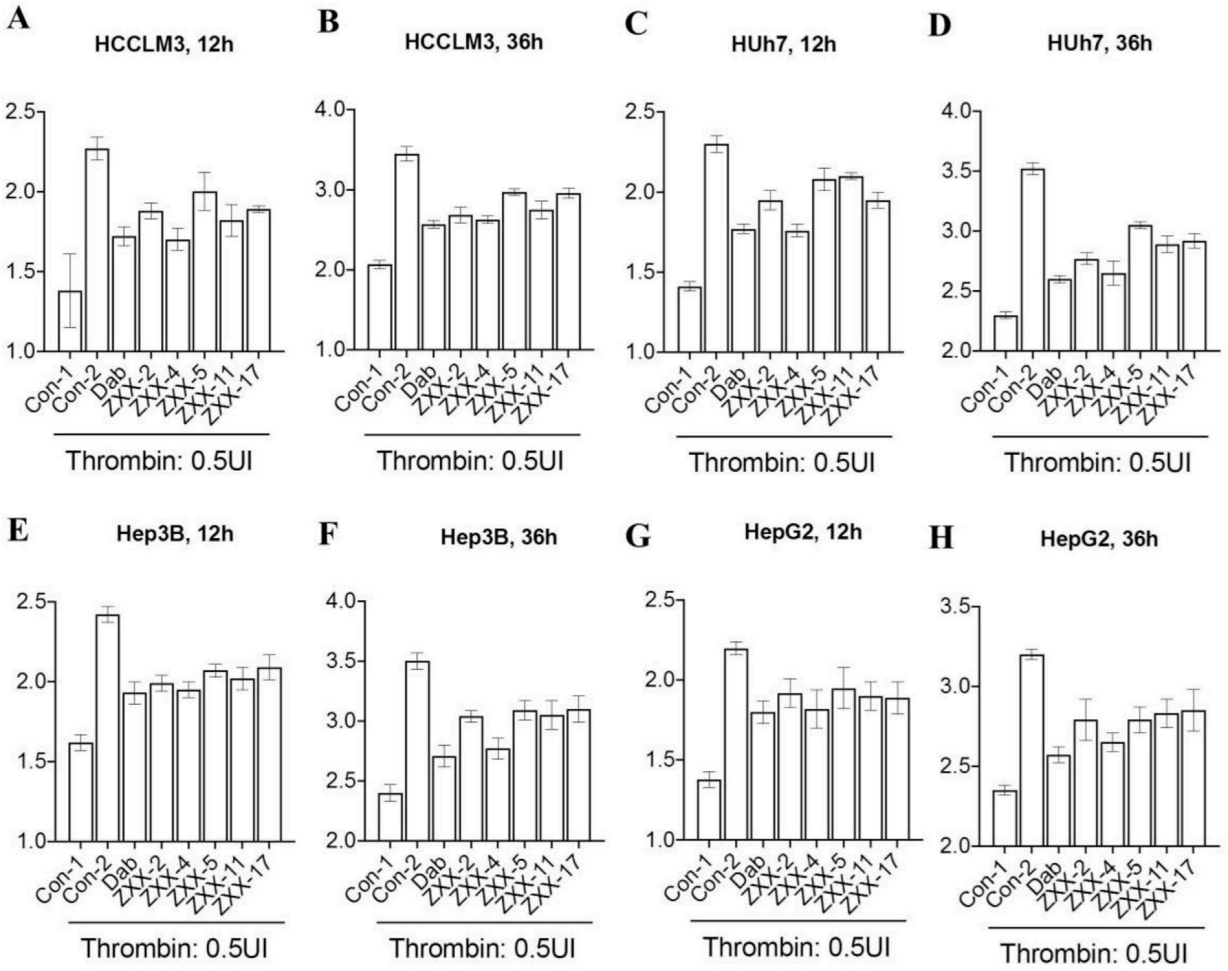
Effect of thrombin inhibitors on the proliferation of HCC cells. **(A, B)** The effect of thrombin inhibitors on the proliferation rate of HCCLM3 cells after 12 and 36 h of treatment *in vitro*; **(C, D)** the effect of thrombin inhibitors on the proliferation rate of Huh 7 cells after 12 and 36 h of treatment *in vitro*; **(E, F)** the effect of thrombin inhibitors on the proliferation rate of Hep3B cells after 12 and 36 h of treatment *in vitro*; and **(G, H)** the effect of thrombin inhibitors on the proliferation rate of HepG2 cells after 12 and 36 h of treatment *in vitro*. Concentration of drugs: 0.5 μM. Data were the mean value of three duplicated experiments.

Thrombin is distributed in plasma, and there is no thrombin detected in tumor cells cultured *in vitro*. Consequently, it is impossible to evaluate the IC_50_ of thrombin inhibitors at the cellular level. Studies have shown that addition of exogenous thrombin dose-dependently accelerated the proliferation of liver cancer cells (using my paper as a reference). Effects of the 5 compounds on the growth rate of tumor cells were evaluated using 4 liver cancer cell lines treated with 0.5 U/mL thrombin, and the results (Figures 6A–H) showed that the proliferation rate of all tumor cell lines was significantly increased after treated with thrombin in the Con-2 group compared with that in the Con-1 group, which verified the promotion effect of thrombin on HCC. The thrombin-induced accelerated proliferation of these tumor cells was reduced to different degrees after treatment with 0.5 μM ZXX-2, ZXX-4, ZXX-5, ZXX-11, ZXX-17 and the positive control dabigatran etexilate. However, the proliferation rate of cells in each treatment group was higher than that in the Con-1 group with no thrombin added, indicating that these compounds had no direct killing effect on the tumor. The activity of the 5 compounds was better at 36 h than that at 12 h. ZXX-4 was the compound with the highest activity, which was comparable to dabigatran etexilate. Therefore, ZXX-4 was selected to evaluate the therapeutic effect on HCC in nude mice.

### 3.4 Evaluation of the efficacy on HCC in BALB/c nude mouse

**FIGURE 7:**
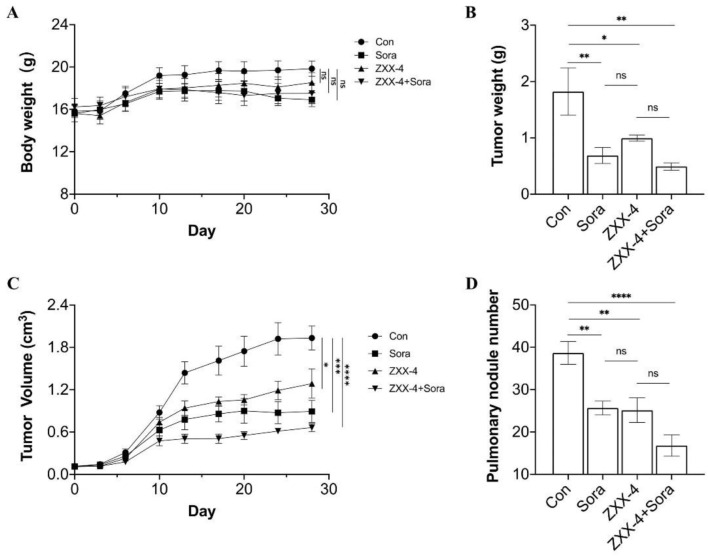
ZXX-4 inhibited HepG2 tumor growth and enhanced the efficacy of sorafenib. HepG2 cells were injected into the inguinal mammary fat pad of BALB/c nude mice, with 6 mice in each group. ZXX-4 (50 mg/kg/day) and/or sorafenib (30 mg/kg/day) were orally administered for 28 days. **(A)** The body weight, **(B)** the tumor weight, **(C)** the tumor volume, and **(D)** the metastatic nodules on the lung surface detected by Indian ink staining of the lung in mice. Data were presented as mean ± S.E.M., and *p*-values were determined by one-way analysis of variance (ANOVA). *p < 0.05, **p < 0.01, ***p < 0.001, and ****p < 0.0001. The *p*-values were analyzed by Pearson’s chi square test using extreme limiting dilution analysis (ELDA) software.

Dosage of ZXX-4 in BALB/c nude mice in pharmacological studies was explored, and it was found that the animals survived 24 h after a single oral administration of 500 mg/kg ZXX-4 with normal diet and movement. Therefore, 50 mg/kg was determined as the dose for pharmacodynamic studies. Sorafenib was used as a positive control and combination treatment. Medication was initiated when the primary tumor was palpable in mice, and administration continued for 28 days. The body weight of the mice was shown in Figure 7A. No statistical difference in the body weight was observed among groups. The body weight of the ZZX-4 group was increased compared with that of the sorafenib group, and the body weight of the ZZX-4 + sorafenib group was increased compared with that of the sorafenib group. As shown in Figure 7B, the tumor weight was significantly reduced in the three treatment groups compared with that of the blank control groups. The efficacy of sorafenib was slightly better than that of ZXX-4, and the tumor weight was further reduced in the ZXX-4 + sorafenib group. The tumor volume curve of each group was shown in Figure 7C, and it was found that the growth rate of tumor volume was decreased in the three treatment groups compared with that in the blank control group, and the findings of tumor volume was consistent with those of the tumor weight. HepG2 tumor cells easily spread to the lungs of mice. The number of lung metastatic nodules in each group was quantitatively evaluated, and it was shown in Figure 7D that the number of lung metastatic nodules in each treatment group significantly decreased, and the efficacy of ZXX-4 was com-parable to that of sorafenib. In addition, the efficacy of sorafenib was enhanced by ZXX-4.

## 4 Conclusion

Currently, the prognosis of HCC is poor, and existing drugs mainly include multitarget tyrosine kinase inhibitors and immune checkpoint inhibitors. The treatment options are limited, and therapeutic targets for HCC with different molecular mechanisms are urgently needed to improve the prognosis of these patients. Studies in recent years have shown that thrombin is associated with the prognosis of HCC, and dabigatran etexilate inhibits thrombin and prevents the proliferation and metastasis of HCC at both cellular and animal levels.

Thrombin inhibitors with novel skeletons were searched in the present study. Firstly, a virtual screening model was established based on the reported interaction conformation of thrombin and dabigatran and key amino acid sites. Structures with a high score were obtained, and its inhibitory activity on thrombin, inhibitory effect on coagulation and preventive ability on HCC proliferation at the cellular level were evaluated. It was found that the activities of compound [Sar1, Ile8]-angiotensin II acetate (ZXX-4) were equivalent to or slightly weaker than those of dabigatran etexilate in each assay. ZXX-4 was previously reported to activate NADPH and NADH oxidases, and the inhibitory activity of compounds with this type of structure on thrombin was discovered for the first time in the present study. Further *in vivo* pharmacodynamic studies showed that ZXX-4 inhibited tumor proliferation, reduced tumor metastasis in nude mice, and enhanced the efficacy of sorafenib. Meanwhile, the above results further confirmed the therapeutic value of thrombin inhibition on HCC. In the future, ZXX-4 can be further explored as an antitumor lead compound with a new skeleton, and inhibition of thrombin can serve as a potential strategy for the treatment of HCC.

## Data Availability

The raw data supporting the conclusions of this article will be made available by the authors, without undue reservation.
